# Caspase-4 disaggregates lipopolysaccharide micelles via LPS-CARD interaction

**DOI:** 10.1038/s41598-018-36811-4

**Published:** 2019-01-29

**Authors:** Jinsu An, Seong Ho Kim, Dohyeon Hwang, Kyung Eun Lee, Min Jung Kim, Eun Gyeong Yang, So Yeon Kim, Hak Suk Chung

**Affiliations:** 1 Center for Theragnosis, Biomedical Research Institute, Korea Institute of Science and Technology, Seoul, 02792 Republic of Korea; 2 Division of Bio-Medical Science & Technology, KIST School, Korea University of Science and Technology, Seoul, 02792 Republic of Korea; 3 Advanced Analysis Center, Korea Institute of Science and Technology, Hwarangno 14-gil 5, Seongbuk-gu, Seoul, 02792 Republic of Korea; 4 Department of Biological Sciences, Sookmyung Women’s University, Seoul, Korea

## Abstract

Lipopolysaccharides (LPS) are a major component of the outer membrane of Gram-negative bacteria and are pathogen-associated molecular patterns recognized by the TLR4/MD2 complex that induces an inflammatory response. Recently, the cytosolic receptors caspase-4/-5/-11 that bind LPS inside the cell and trigger inflammasome activation or pyroptosis, have been identified. Despite the important roles of caspase-4 in human immune responses, few studies have investigated its biochemical characteristics and interactions with LPS. Since caspase-4 (C258A) purified from an *Escherichia coli* host forms aggregates, monomeric proteins including full-length caspase-4, caspase-4 (C258A), and the CARD domain of caspase-4 have been purified from the insect cell system. Here, we report the overexpression and purification of monomeric caspase-4 (C258A) and CARD domain from *E*. *coli* and demonstrate that purified caspase-4 (C258A) and CARD domain bind large LPS micelles and disaggregate them to small complexes. As the molar ratio of caspase-4 to LPS increases, the size of the caspase-4/LPS complex decreases. Our results present a new function of caspase-4 and set the stage for structural and biochemical studies, and drug discovery targeting LPS/caspase-4 interactions by establishing a facile purification method to obtain large quantities of purified caspase-4 (C258A) and the CARD domain.

## Introduction

**Figure 1:**
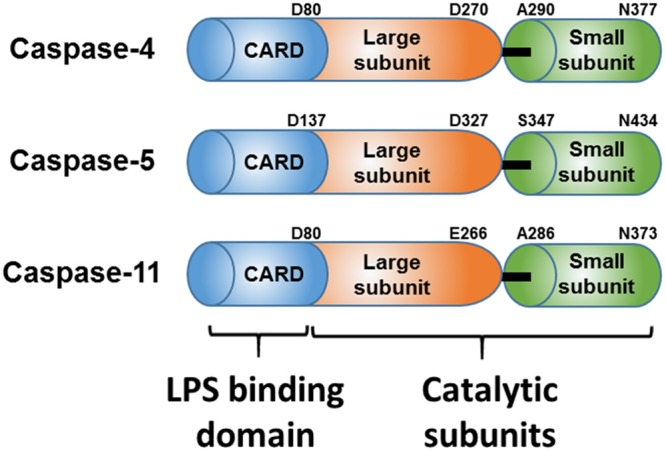
Schematic domain structures of the cytosolic LPS receptors Caspase-4, Caspase-5 and Caspase-11. The amino acid numbering above corresponds to that in the UniProt database^16^.

Lipopolysaccharides (LPS) are a major component of the outer membrane of Gram-negative bacteria. Their structure comprises three major parts, lipid A, a core oligosaccharide, and the O-antigen. Lipid A contains two glucosamine units with hexa-acyl chains and two phosphate groups, and is referred to as an active unit that causes the inflammatory response in mammalian hosts^1^. LPS is also referred to as an endotoxin, and is a representative pathogen-associated molecular pattern recognized by the host innate immune system. LPS micelles in humans are recognized by the LPS binding protein (LBP), and LPS is transferred to CD14 and forms a heterodimeric complex with TLR4/MD2 on the surface of immune cells^2–4^. While the complex formation initiates signal transductions resulting in the expression of genes involved in host defense^3,5^, excessive amounts of LPS can result in a lethal septic shock and even death. Recently, caspase-4/-5 from human and caspase-11 from mouse were reported as cytosolic LPS receptors. Caspase-4/-5/-11 are enzymes belonging to a family of cysteine proteases and are classified as inflammatory caspases. These enzymes are synthesized as a latent zymogen containing the caspase activation and recruitment domain (CARD), and large and small subunits (Fig. 1). The physiological functions of caspase-4/-5/-11 have been recently elucidated. In 2013, Kayagaki *et al*. and Hagar *et al*. independently found that caspase-11 is activated by cytosolic LPS and leads to non-canonical inflammasome activation^6,7^. The next year, Shi and colleagues showed that caspase-4 and caspase-11 directly bind to cytosolic LPS through their CARD domain and suggested that LPS binding induces oligomerization of caspase-4 or caspase-11, and that oligomerization is required for caspase-4 or caspase-11 activation and formation of non-canonical inflammasome^8^. Activated caspase-4 induces inflammasome formation as well as pyroptosis, a form of inflammatory cell death through cleavage of Gasdermin D^9^. Several studies have also investigated how LPS enters cells and demonstrated that outer-membrane vesicles (OMVs) secreted by living Gram-negative bacteria enter the host cell through endocytosis^10^. *Escherichia coli* O111:B4 LPS could bind to the cholera toxin B subunit followed by endocytosis via the GM1 ganglioside^6,7^, or release of bacteria from the vacuoles via small interferon-induced guanylate binding proteins^11,12^. However, the mechanism of LPS recognition by caspase-4 through the CARD domain and the events occurring after binding to LPS at the molecular level are still unclear. Contrary to previous suggestions that caspase-4 is oligomerized by LPS binding^8^, another study suggested that caspase-4 just binds to large LPS micelles that provide a surface for caspase-4 activation resulting in high molecular weight complexes^13^. Since *E*. *coli* has endogenous LPS, full-length caspase-4/-5/-11 purified from *E*. *coli* are isolated as high molecular weight aggregates; therefore, all biochemical studies have been performed with caspase-4 purified from the Bac-to-Bac Baculovirus Expression System^8,13^. Here we established the expression and purification procedures for the monomeric caspase-4 (C258A) variant and its CARD domain from an *E*. *coli* system at the mg scale to understand the roles of caspase-4 during its interactions with LPS. With the purified CARD domain and caspase-4 (C258A), we demonstrate for the first time that these proteins not only bind to large LPS micelles, but also break them into lower molecular weight complexes *in vitro*. We also found that as the molar ratio of caspase-4 to LPS increases, the size of the caspase-4/LPS complex decreases as observed by gel filtration chromatography and transmission electron microscopy (TEM). Our results provide a fundamental basis to understand the role of caspase-4 during non-canonical inflammasome formation by cytosolic LPS, and the availability of *E*. *coli* purified monomeric caspase-4 (C258A) and CARD domain will facilitate further biochemical and structural studies as well as drug discovery for sepsis and inflammation related diseases.

## Results

### Overexpression and purification of recombinant caspase-4 (C258A) and CARD domain using *E. coli* cells

**Figure 2:**
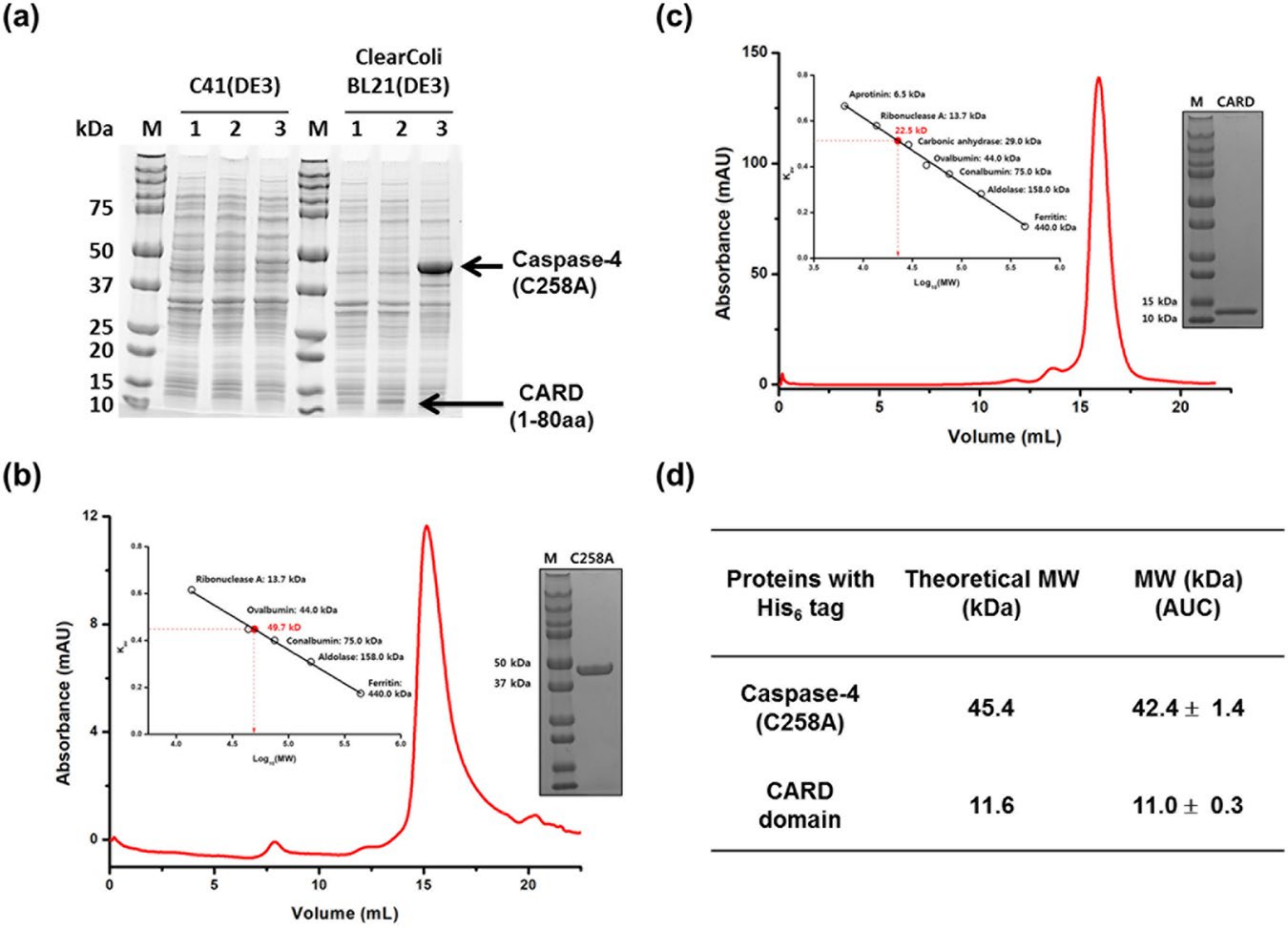
Monomeric caspase-4(C258A) and CARD purified from *E*. *coli* (**a**) A 4–20% SDS-PAGE gel comparing the expression levels of recombinant proteins in two different *E*. *coli* hosts. Whole cell lysates from pET28b empty vector (Lane 1), pET28b harboring the CARD domain of caspase-4 (Lane 2), and pET28b harboring caspase-4 (C258A) (Lane 3). The amount of protein loaded on the SDS gel was normalized to a cell culture biomass based on OD_600_. SEC elution profiles of caspase-4 (C258A) (**b**) and CARD domain (**c**) from Superdex 200 10/300 gl columns calibrated with protein standards and SDS-PAGE gels of the purified caspase-4 (C258A) (**b**) and CARD domain (**c**). 5 µg of purified protein was loaded in each lane. Calibration curves are presented in insets and enlarged calibration curves are presented in Supplementary Fig. S5. (**d**) Molecular weight (MW) determination of purified caspase-4 (C258A) and CARD domain from ClearColi BL21(DE3) by analytical ultracentrifugation (AUC) and theoretical MW. Shown are the mean values ± standard deviation. SDS-PAGE gels shown in **a**, **b**, and **c** were cropped for clarity and full-length gels are presented in Supplementary Figs S1 and S4.

Despite the important roles of caspase-4, biochemical and structural studies on caspase-4 have been limited due to difficulties in purification. Although wild type caspase-4 would provide information regarding caspase-4 activation by LPS, we could not detect intact full-length caspase-4 on an SDS-PAGE gel when we tried to purify wild type caspase-4 in the *E*. *coli* host. Therefore, instead of using wild type caspase-4, we decided to study the LPS/caspase-4 complex using the caspase-4 (C258A) variant and the CARD domain that plays a critical role in LPS recognition. According to a previous study^8^, caspase-4 (C258A), a catalytically inactive variant, purified from *E*. *coli* cells was eluted as about 600 kDa aggregates based on the size exclusion chromatography (SEC), whereas caspase-4 expressed and purified from Sf21 insect cells was a monomer as determined by analytical ultracentrifugation and static light scattering. Since protein purification from insect or mammalian cells is normally more expensive, more cost and time effective methods are required for performing structural and biochemical studies as well as for drug screening targeting the interaction between LPS and the CARD domain of caspase-4. Therefore, we asked how we could establish a purification method for monomeric caspase-4 (C258A) and the CARD domain from *E*. *coli*, which has not been reported before. We then noticed that unlike LPS, lipid IV_A_, a precursor of the LPS biosynthetic pathway, did not activate caspase-4 as much as LPS and induced a lower molecular oligomer than LPS did in a native gel^8^. Therefore, we hypothesized that if *E*. *coli* produces lipid IV_A_ as its only LPS, it may allow expression and purification of caspase-4 and the CARD domain in the monomeric form. To test this, we transformed pET28b-caspase-4 (C258A) and pET28b-CARD into ClearColi BL21 (DE3) cells that produce lipid IV_A_ instead of full-length LPS^14^ and C41 (DE3)^15^ that produces LPS. With two different *E*. *coli* hosts, we compared the expression level of caspase-4 (C258A) and the CARD domain containing 1–80 amino acids of caspase-4, as suggested by UniProt^16^ (Fig. 1). The expression levels of caspase-4 (C258A) from ClearColi BL21(DE3) were much higher than those in C41 (DE3) under the same conditions (Fig. 2a). In addition, the CARD domain was rarely expressed in C41 (DE3), but was well expressed in ClearColi BL21 (DE3) based on an SDS-PAGE gel (Figs 2a and S1). When we purified Caspase-4 (C258A) and CARD domain from ClearColi BL21 (DE3), the proteins were eluted at ~600 kDa from a gel filtration column (Supplementary Fig. S2), implying that caspase-4 still binds to lipid IV_A_ micelles maybe with lower affinity compared to LPS. Therefore, to obtain monomeric caspase-4 (C258A) or CARD domain, we had to design a method to dissociate the caspase-4/lipid IV_A_ interaction. Shi *et al*.^8^ reported that when Tween 20 was present, caspase-4 did not form a high molecular weight oligomer with LPS in a native gel. It is highly possible that caspase-4 expressed in the cytoplasm of *E*. *coli* may bind to LPS during lysis, so if we lysed cells in the presence of 1% Tween 20, caspase-4 could not form complexes with lipid IV_A_, thereby resulting in monomeric proteins. Based on this hypothesis, we added 1% Tween 20 to the lysis buffer and removed it during Ni-NTA affinity chromatography, and the resulting caspase-4 (C258A) was eluted at ~49.7 kDa from a gel filtration column (Supplementary Fig. S2) and monomeric caspase-4 (C258A) was also determined via analytical ultracentrifugation (Fig. 2b,d). The purity of caspase-4 (C258A) was determined by SDS-PAGE (Supplementary Fig. S3). We noticed that after Ni-NTA affinity chromatography, two major protein fractions were purified for caspase-4 (C258A). The lower molecular weight fraction in lane 4 of Supplementary Fig. S3a may be attributed to the cleaved N-terminal domain of caspase-4 containing a His_6_-tag by *E*. *coli* endogenous protease as caspase-4 (C258A) is a catalytically inactive variant. This endogenous protease present in *E*. *coli* and the auto-cleavage activity of caspase-4 may contribute toward the difficulty in expressing intact full-length caspase-4 in *E*. *coli* in the soluble form. Since we found that the CARD protein exhibited heat stability, we could purify CARD protein by treating the cell lysate for 30 minutes at 70 °C. After heat treatment, the Ni-NTA affinity chromatography rendered about >95% pure protein based on SDS-PAGE (Supplementary Figs S3 and S4). While the CARD domain was eluted at a molecular weight of ~22.5 kDa by gel filtration chromatography (Figs 2c and S5), the molecular weight of the CARD domain was determined as a monomer via analytical ultracentrifugation (Fig. 2c and d). With the described method, we could obtain about ~5 mg and 4 mg of the CARD domain and Caspase-4 (C258A) from 1 L *E*. *coli* culture, respectively. This is the first example of full-length caspase-4 (C258A) and the CARD domain being expressed and purified from *E*. *coli* cells in the monomeric form.

### Complex sizes of caspase-4 (C258A/LPS) or CARD/LPS depend on the molar ratio between caspase-4 (C258A) and LPS or CARD and LPS

**Figure 3:**
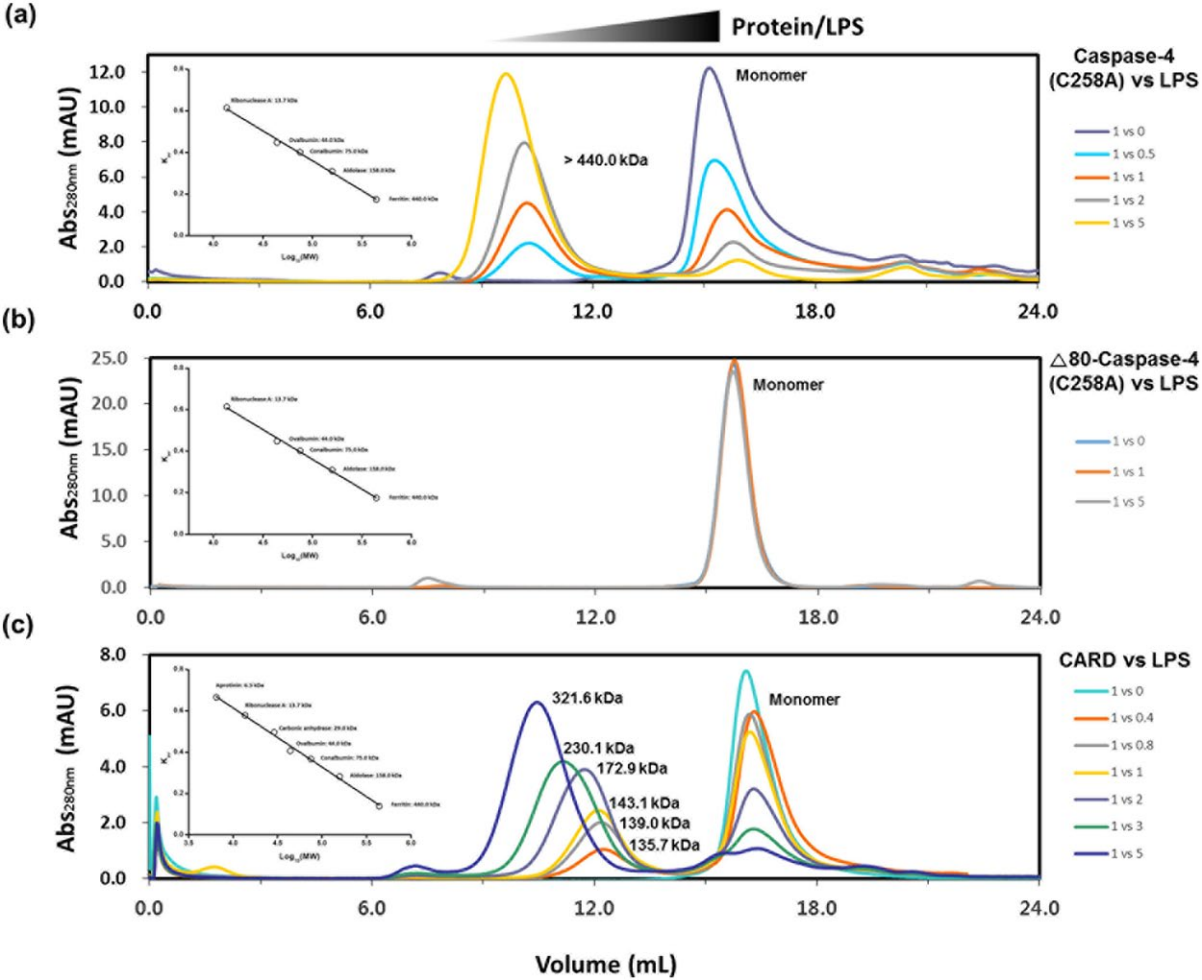
*E*. *coli* purified caspase-4(C258A) and CARD form LPS/protein complexes in a molar ratio dependent manner. SEC elution profiles of different molar ratios of Ra-LPS/Caspase-4 (C258A) (**a**), Ra-LPS/Δ80 caspase-4 (C258A) (**b**), and Ra-LPS/CARD domain complex (**c**) from Superdex200 10/300 gl columns calibrated with protein standards. Calibration curves are presented in insets and enlarged calibration curves are presented in Supplementary Fig. S6.

After purification of caspase-4 (C258A) and the CARD domain from *E*. *coli*, we wanted to test whether these proteins have the ability to bind LPS as previously reported for caspase-4 purified from sf1 cells^8,13^. To identify complex formation and the relative size of the complexes, we incubated fixed concentrations of caspase-4 (C258A) (12.5 μM) or CARD domain (23 μM) with an increased amount of Ra-LPS composed of lipid A and inner- and outer-core polysaccharides but no O-antigen region. After incubation, the reaction mixtures were injected into a gel filtration column, and the retention times for the samples were compared. As shown in Fig. 3a, purified caspase-4 (C258A) formed higher molecular weight complexes as the LPS amounts were increased. These complexes were abolished if the CARD domain was deleted (Δ80 caspase-4(C248A)) (Fig. 3b). When we looked at the data in detail, we noticed several interesting observations. First, LPS/Caspase-4 (C258A) formed a ~600 kDa complex, and we did not observe a lower molecular weight complex even though enough caspase-4 remained in a monomeric form. Second, to ensure that all monomeric caspase-4 participated in the complex formation, 2–5 molar equivalent of Ra-LPS was required. This implied that the LPS and caspase-4 complex does not form in a 1:1 stoichiometry. Third, considering that the monomeric molecular weights of caspase-4 (C258A) and Ra-LPS are 45.4 kDa and ~3.9 kDa, respectively and based on the rough stoichiometry we observed (1:2–5 = caspase-4 (C258A): Ra-LPS), about 9–10 molecules of caspase-4 (C258A) might be participating in the complex formation. These phenomena were also observed when we monitored the CARD domain and the Ra-LPS complex formation, as shown in Fig. 3c. For the CARD/Ra-LPS complex, the first CARD/LPS complex in Fig. 3c was eluted at ~136 kDa. Since the Superdex 200 10/300 GL column has an optimum separation range from 10 kDa to 600 kDa (Supplementary Fig. S6), we noticed a gradual increase in the complex as the concentration of Ra-LPS was increased. Intriguingly, we observed a discrete and symmetric UV trace from each complex regardless of the molar ratio between the proteins/Ra-LPS. (Fig. 3a,c). This implies that caspase-4/Ra-LPS form a complex of a specific size depending on the molar ratio rather than on non-specific micelles. Our results demonstrate that caspase-4 (C258A) and the CARD domain purified from *E*. *coli* bind to LPS micelles and that the 1–80 amino acid region of caspase-4 is enough to recognize LPS. Notably, the minimum concentration of Ra-LPS used in this study was 6.25 μM. Even considering the dilution effect during gel filtration chromatography (24 mL column volume), the minimum Ra-LPS concentration we used was about ~130 nM. According to a previous study, the hydrodynamic radius of Kdo_2_-lipid A that is the core structure of Ra-LPS is nearly 80 nm at a concentration above CMC (41.2 ± 1.6 nM) at 25 °C^17^, and the hydrodynamic diameter of the 2000 kDa dextran was calculated as 54.4 nm^18^. Therefore, if CARD simply binds to the surface of Ra-LPS micelles, the complexes would be eluted in a void volume of the column rather than being eluted at 136–600 kDa, as observed in Fig. 3. The results imply that caspase-4 does not simply bind to a large LPS micelle; rather, caspase-4 reduces the size of the LPS micelle.

### Caspase-4 (C258A) disaggregates large LPS micelles to smaller complexes via its CARD domain

**Figure 4:**
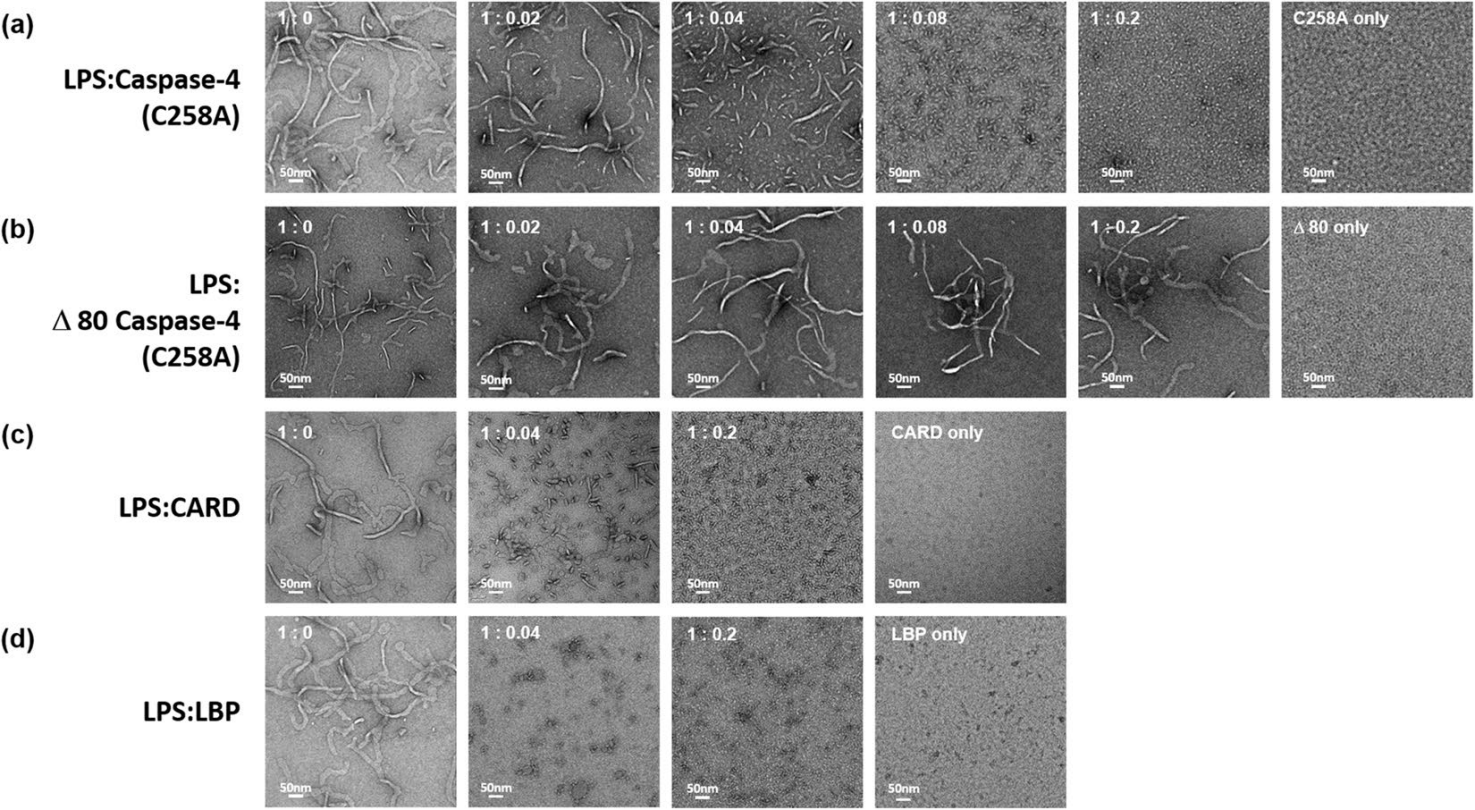
Caspase-4 disaggregates large LPS micelles to smaller LPS/protein complexes via the CARD domain. TEM images of different molar ratios of Ra-LPS/caspase-4 (C258A) (**a**), Ra-LPS/Δ80 caspase-4 (C258A) (**b**), Ra-LPS/CARD (**c**), and Ra-LPS/LBP (**d**) were visualized by negative-staining. Scale bar represents 50 nm.

The above implication raises the following questions. Does caspase-4 bind large LPS micelles and disaggregate them to smaller LPS/caspase-4 complexes? If the molar ratio of Ra-LPS and caspase-4 determines the size of the complex, are large LPS micelles broken into smaller complexes when we add an increased amount of caspase-4 into a fixed amount of Ra-LPS micelles? To answer these questions, 23 μM of Ra-LPS was titrated with increased amounts of caspase-4 (C258A) or the CARD domain and analyzed using a transmission electron microscope (TEM). Based on the TEM images, we observed that large LPS micelles were dramatically disaggregated into smaller complexes by adding 0.92 μM of caspase-4 (C258A) or the CARD domain corresponding to a 1:0.04 molar ratio of the Ra-LPS:protein (Fig. 4a,c). As the amount of proteins increased, the size of the Ra-LPS/protein complex was decreased. This disaggregation ability of caspase-4 (C258A) is reminiscent of LBP protein^2^ that we used as a positive control in our TEM experiment (Fig. 4d). We also observed that disaggregation of LPS micelles by caspase-4 (C258A) completely relied on the presence of the CARD domain (Fig. 4b,c).

### Direct binding between caspase-4 purified from *E. coli* and LPS

**Figure 5:**
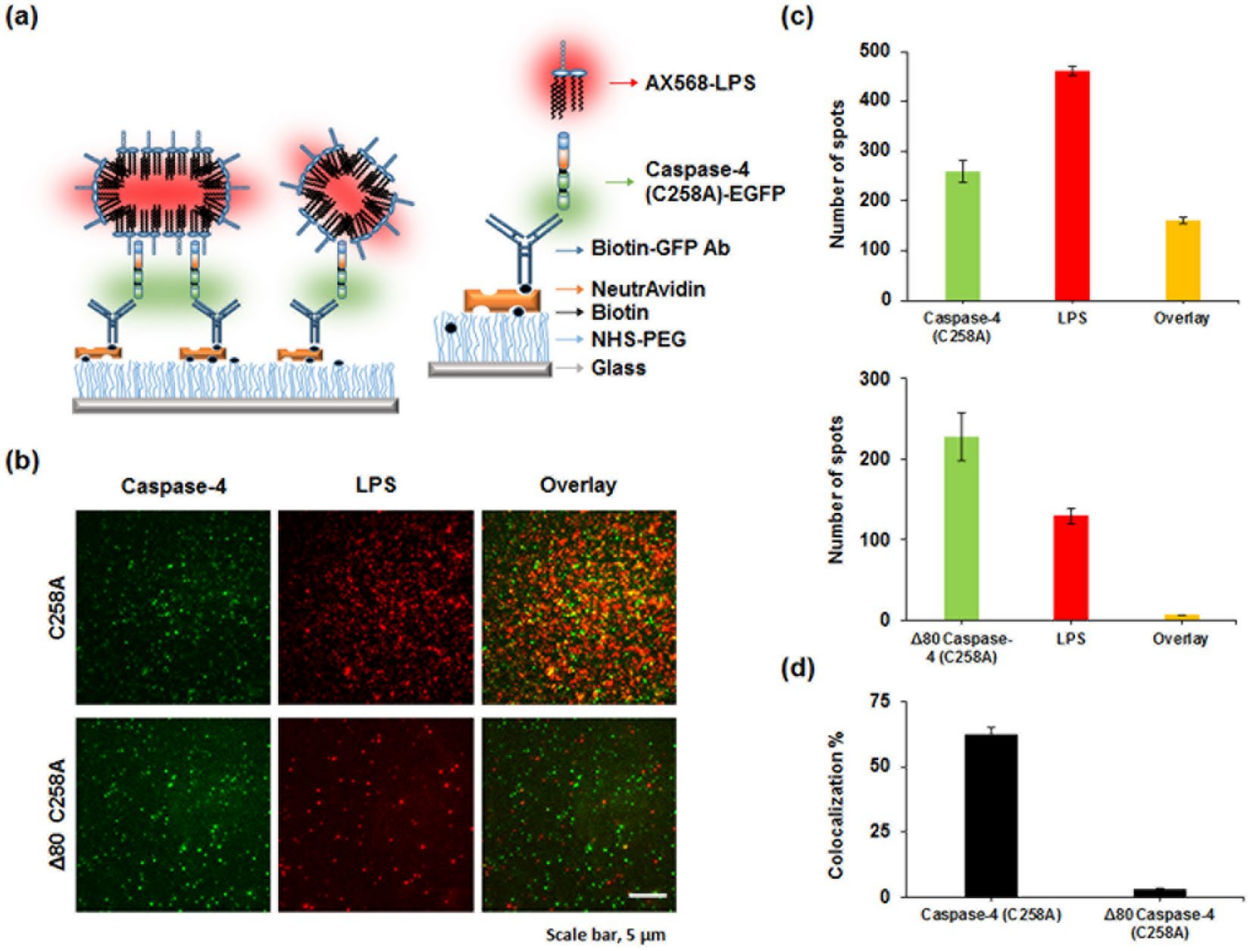
Single-molecule co-localization between EGFP tagged caspase-4 (C258A) (or Δ80 caspase-4 (C258A)) and AX568-LPS. Caspase-4 (C258A)-EGFP (or Δ80 caspase-4 (C258A)) and AX568-LPS were imaged after immobilization of 100 pM of Caspase-4 with anti-GFP followed by incubation with 6 µM LPS in the presence of 1 µM of AX568-LPS. (**a**) Schematic of the single-molecule co-localization assay and surface modification. (**b**) Upper panel: Representative images (25 × 25 µm) for caspase-4 (C258A)-EGFP (left) and AX568-LPS (middle), and their overlapped images (right). Lower panel: Representative images (25 × 25 µm) for Δ80 caspase-4 (C258A)-EGFP (left) and AX568-LPS (middle), and their overlapped images (right). Scale bar, 5 µm. (**c**) Average number of fluorescent spots for caspase-4 (green), LPS (red), and co-localized caspase-4 (C258A) (or Δ80 caspase-4 (C258A)) and LPS (orange) per image. ± indicates standard error of mean (SEM). (**d**) Average co-localization efficiency was determined by calculating the number of co-localized spots divided by the number of caspase-4 (C258A) (or Δ80 caspase-4 (C258A)) spots.

Although purified caspase-4 and Ra-LPS seems to form stable complexes in size exclusion chromatography (Fig. 3), we performed single molecule co-localization experiments using total internal reflection fluorescence microscopy (TIRF) to examine direct interactions between caspase-4(C258A) and Ra-LPS. To visualize caspase-4 (C258A) and Ra-LPS by fluorescence, we purified caspase-4 (C258A) or Δ80 caspase-4 (C258A) conjugated with Enhanced Green Fluorescent Protein (EGFP) at its C-terminus (Supplementary Fig. S7). EGFP conjugated proteins were immobilized on a polyethylene glycol (PEG) coated glass surface using anti-GFP antibodies. After immobilization of caspase-4 (C258A), we introduced Alexa Fluor 568 (AX568) labeled LPS (AX568-LPS) (Fig. 5a). While similar numbers of caspase-4 (C258A) or Δ80 caspase-4 (C258A) protein were immobilized on the surface (Fig. 5b,c), the percentile of co-localized AX568-LPS with caspase-4 (C258A) and Δ80 caspase-4 (C258A) was dramatically reduced from 62.4 ± 2.7% to 2.9 ± 0.6% (Fig. 5b–d), respectively. From these results, we could establish the direct binding between caspase-4 (C258A) purified from *E*. *coli* and LPS as well as the role of CARD domain.

## Discussion

**Figure 6:**
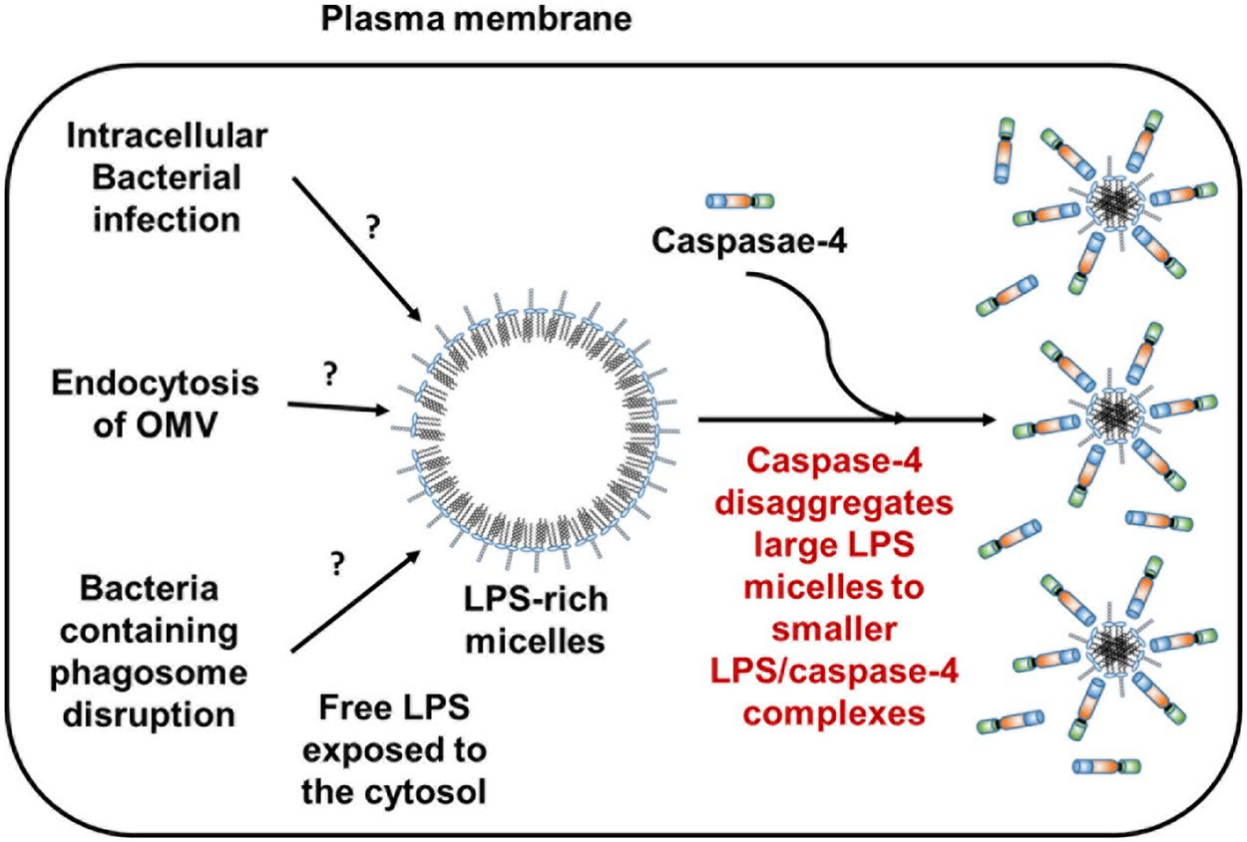
Proposed role of caspase-4 in response to LPS-rich micelles exposed to cytosol. Caspase-4 not only binds LPS-rich micelles but also disaggregates them to smaller LPS/caspase-4 complexes.

Caspase-4 is known as a receptor for intracellular LPS, and induces non-canonical inflammasome formation and pyroptosis. Numerous biological studies related to caspase-4 have found that it plays roles in acute and chronic diseases such as sepsis and cancer^6,7,19^. Accordingly, targeting caspase-4/LPS interactions is important for drug development. However, few studies^8,13^ have sought to understand the biochemical characteristics of caspase-4 or the CARD domain as an LPS receptor *in vitro*. Biochemical, structural, and pharmaceutical studies require a facile method to obtain purified caspase-4 or the CARD domain in large quantities. In this study, we have established an efficient expression and purification system for full-length caspase-4 (C258A) and its CARD domain using *E*. *coli* cells. With purified caspase-4 (C258A) and CARD, we showed that these proteins bind to LPS micelles as well as break large LPS micelles into smaller LPS/protein complexes in an LPS/protein molar ratio-dependent manner (Figs 3, 4 and 6). We also showed that caspase-4 disaggregates LPS equally well regardless the presence of the O-antigen region (wild type *E*. *coli* O55:B5 LPS containing O-antigen vs. Ra-LPS without O-antigen, Supplementary Fig. S8). Altogether, we found that caspase-4 has a similar function to LBP that binds and disaggregates LPS micelles depending on the molar ratio between LBP and LPS during the LPS transfer cascade from LBP/CD14 to TLR4/MD2^2,4^. Until the discovery of caspase-4 as a cytosolic LPS receptor, the LBP/CD14/TLR4-MD2 system was representative of the LPS recognition system in mammalian hosts. LBP binds LPS and then CD14 binds to LPS-bound LBP and induces changes in LBP structure. The structural change transfers LPS from LBP to CD14, and LPS is eventually transferred from CD14 to MD2 and forms the LPS/TLR4/MD2 complex^4^. The LPS/TLR4/MD2 complex induces intracellular signaling pathways. During the LPS transfer process, large LPS micelles are effectively disaggregated when LBP and CD14 are presented together^4^ or when the molar ratio of LBP to LPS is increased^2^. Previously, two different explanations were suggested for high molecular weight entities observed by pore-limited native gel analysis and gel filtration chromatography for LPS and caspase-4/-5/-11 recognition. One study suggested that LPS binding induces caspase-4 oligomerization to large molecular entities^8^ and activates caspase-4. On the other hand, another study suggested that caspase-4 binds to LPS aggregates or LPS-rich membrane interfaces on which caspase-4 can assemble in the appropriate proximity or orientation to active caspase-4^13^. In contrast to previous suggestions, we have found that caspase-4 binds large LPS micelles, and disaggregates them to smaller LPS/caspase-4 complexes (Fig. 4), which may provide the appropriate proximity and orientation to active caspase-4. According to a recent study, the expression level of Guanylate Binding Protein (GBP) is increased by OMV stimulation. This study also confirmed that GBP binds to LPS molecules on the surface of the OMV, which facilitates LPS recognition by caspase-11^20^. While it is not currently clear whether caspase-4 itself has the full ability to extract a single LPS molecule from the OMV or bacteria, large LPS micelles in the cytosol formed from the OMV or by the lysis of whole bacterial cells by GBP would be recognized by caspase-4 and are disaggregated to smaller LPS/caspase-4 complexes. Thus, caspase-4 and GBP seem to have similar functions carried out by the LBP/CD14/TLR4/MD4 system for LPS-dependent signaling, including binding to LPS, disaggregating LPS micelles, and activating downstream signaling cascades. So far several proteins including LBP^2^, albumin, hemoglobin, and high-density lipoprotein have been reported to possess disaggregating ability for LPS micelles^21^.

Still many questions remain to be answered to understand LPS/caspase-4 recognition, such as whether different sizes of LPS/caspase-4 complexes induce different levels of downstream effects including protease activity of caspase-4, inflammation activation, and pyroptosis; how caspase-4 binds to LPS at the molecular level; and whether LPS binding induces structural changes in caspase-4 or not. We have now set the stage to study some of these questions by establishing large quantities of purified caspase-4 (C258A) and the CARD domain. While caspase-4 has been known to recognize and bind LPS using the CARD domain, we found a fundamental role of caspase-4 that disaggregates LPS micelles to form stable LPS/caspase-4 complexes in LPS dependent signaling implying that each cell may form different sizes of LPS/caspase-4 complexes depending on the caspase-4 expression levels and the amount of LPS exposed to the cytosol of the cell during infection.

## Experimental Procedures

### Materials

ClearColi BL21(DE3) were purchased from Lucigen (USA). All PCR amplification executed using KOD Hot Start DNA Polymerase (Novagen, USA) or Pfu polymerase (ELPiS, ROK) and a T3000 thermocycler (Biometra). Sequencing and primer synthesis were done at Macrogen (Seoul, ROK). His-tag human LBP protein purified from HEK293 was purchased from Sino Biological. Ra-LPS mutant WT-LPS- from *E*. *coli* O55:B5 and all other chemicals were purchased from Sigma-Aldrich (Korea) unless stated otherwise.

### Cloning of the CASP4 variants

The codon optimized CASP4 gene encoding for a human caspase-4 protein was synthesized by Integrated DNA Technology (Coralville, USA) and amplified by PCR using forward primer (5ʹ- AGG TCG TCA TAT GGC TGA GGG TAA TCA TTC G-3ʹ) and reverse primers (5ʹ-CCG CAA GCT TTC AAT TAC CCG GAA AAA GAT AGA AAT ACC-3ʹ) for full length caspase-4 and (5ʹ-GCA GAA GCT TTC AGT TCG GAT GCG CTT TCT TG-3ʹ) for CARD domain, respectively. Genes were inserted into pET28b (+) using NdeI and HindIII restriction sites. Catalytically inactive caspase-4 (C258A) variant and CARD (1–80aa) truncated constructs (Δ80 caspase-4 (C258A))were generated by QuikChange site-directed mutagenesis following manufacture’s protocol (Agilent, USA). For EGFP fusion proteins, amplified EGFP gene was inserted at C-terminus of pET28b-caspase-4(C258A) and pET28b-Δ80 caspase-4 (C258A) using HindIII and XhoI restriction sites. Recombinant plasmids were transformed in C41(DE3) or ClearColi BL21(DE3) by TSS method^22^.

### Expression of Caspase-4 (C258A) and CARD domain from *E. coli* cells

Single colony from C41(DE3)/pET28b-caspase-4(C258A), ClearColi BL21(DE3)/pET28b-caspase-4(C258A), C41(DE3)/pET28b-CARD, or ClearColi BL21(DE3)/pET28b-CARD was inoculated in 10 mL of LB media containing kanamycin (50 μg/mL) and cultured during overnight at 30 °C. 1 L of LB medium containing kanamycin (50 μg/mL) was inoculated with 10 mL of the overnight culture, and grown at 30 °C. When OD_600_ reached at about 0.25, temperature of the culture was lower to 18 °C and proteins were induced with 0.2 mM isopropyl β-d-1-thiogalactopyranoside at OD_600_~0.6 and were expressed at 18 °C for 16–18 hours. 1 mL of each culture, which is equivalent to OD_600_ = 1.0 was harvested and re-suspended with 100 μL of 1 × Laemmli protein sample buffer and 10 μL of the samples were loaded in each lane of the SDS-PAGE gel (Fig. 2a).

### Purification of human caspase-4 (C258A), Δ80 caspase-4 (C258A), and EGFP conjugated caspase-4 (C258A) and Δ80 caspase-4 (C258A)

Cell pellets from 1 L LB culture were suspended with 30 mL of buffer A (20 mM Tris-HCl (pH 7.9), 300 mM NaCl, 20 mM imidazole, 5 mM beta-mercaptoethanol (BME), 1% Tween20) supplemented with a tablet protease inhibitor cocktail (complete, EDTA-Free, Sigma). Cells were lysed by sonication. Soluble fractions were separated by centrifugation (20,000 × g for 40 min at 4 °C). The supernatant was loaded on a pre-equilibrated 1 mL HisTrap column (GE Healthcare, USA) with buffer B (20 mM Tris-HCl (pH 7.9), 300 mM NaCl, 20 mM imidazole) and washed with 10 column volumes (CV) of buffer B and 50 CV of buffer C (20 mM Tris-HCl (pH 7.9), 300 mM NaCl, 50 mM imidazole), and eluted with buffer D (20 mM Tris-HCl (pH 7.9), 300 mM NaCl, 300 mM imidazole). Eluent was injected to HiLoad 26/600 superdex 200 pg (GE Healthcare, USA) column equilibrated with buffer E (20 mM HEPES pH 7.5, 300 mM NaCl, 10% Glycerol, 0.5 mM TCEP). The major fractions were analyzed with 4–20% SDS-PAGE Gel stained with coomassie brilliant blue. Protein concentrations were determined by Bradford protein assay kit II (Bio-Rad, USA) with bovine serum albumin as the standard.

### Purification of human caspase-4 CARD domain

Cell pellets were suspended with 30 mL of buffer B. Cells were lysed by sonication and soluble fractions were separated by centrifugation (20,000 × g for 40 min at 4 °C). The supernatant was heated at 70 °C for 30 min and spun down at 20,000 × g for 30 min to remove denatured proteins. The resulting supernatant was loaded on a pre-equilibrated 1 mL HisTrap column (GE Healthcare, USA) with buffer B and washed with 10 CV of buffer B and 10 CV of buffer B containing 0.1% Tween20 and 50 CV of buffer C, and eluted with buffer D. Eluent was injected to HiLoad 26/600 superdex 200 pg (GE Healthcare, USA) column equilibrated with a buffer F (20 mM HEPES pH 7.5, 300 mM NaCl). Protein purity and concentration were determined by method described above.

### Analytical Ultracentrifugation

Sedimentation velocity analysis was carried out with a ProteomeLab XL-A (Beckman Coulter) with an AN 60 Ti rotor at 20 °C. Caspase-4 (C258A) and CARD domain were prepared in a buffer E at a 1.7 mg/mL or in a buffer F at a 1.5 mg/mL, respectively. Each buffer for each protein was used as the reference solution. Data were collect at a speed of 42,000 rpm. Absorbance scans were measured at 280 nm at intervals of 20 min in a radial direction. The molecular weights were calculated by the SEDFIT software.

### Size exclusion chromatography (SEC) of Ra-LPS and protein mixture

Final 12.5 μM of purified caspase-4 (C258A) and Δ80 caspase-4 (C258A) were incubated with 0, 6.25, 12.5, 25, and 62.5 µM or 0, 12.5 and 62.5 µM of Ra-LPS respectively. And 23 μM of purified CARD domain was incubated with 0, 9.2, 18.4, 23, 46, 69, and 115 µM of LPS Ra mutant. The molecular weight of Ra-LPS was estimated as 3,835 g/mole. Final volume was set in 600 μL scale with buffer F for CARD/Ra-LPS and buffer G (20 mM HEPES (pH 7.5), 300 mM NaCl, 0.5 mM TCEP) for caspase-4 (C258A) or Δ80 caspase-4 (C258A)/Ra-LPS and incubated at 37 °C for 30 min. Samples were centrifuged (20,000 × g, 10 min, 4 °C) and supernatant was injected to a superdex 200 10/300 gl column equilibrated with buffer F for CARD/Ra-LPS sample and buffer G for caspase-4(C258A) or Δ80 caspase-4 (C258A)/Ra-LPS.

### Transmission electron microscopy

Complexes of LPS (23 µM) and caspase-4 (C258A), Δ80 caspase-4 (C258A), CARD domain or LBP were reconstituted at molar ratio of 1:0, 1:0.02, 1:0.04, 1:0.08 or 1:0.2 and loaded these onto glow-discharged carbon-coated grids, rinsed and stained them with 2% (w/v) uranyl acetate. We recorded images using a Tecnai F20 field emission gun electron microscope operated at 200 kV.

### Single-molecule co-localization experiments

Protein immobilization and single-molecule co-localization setup was utilized as reported previously^23^. Briefly, biotin labeled GFP antibody (abcam) was immobilized on a microfluidic chamber-assembled, PEG-coated coverslip. 100 pM of either EGFP-conjugated caspase-4 (C258A) (Caspase-4 (C258A)-EGFP) or EGFP-conjugated caspase-4 (C258A) lacks CARD domain (Δ80 Caspase-4 (C258A)-EGFP) was added to the treated surface and washed with the buffer G. Then, 6 µM of AX568 labeled *E*. *coli* O55:B5 lipopolysaccharide (Thermo, USA) (AX568-LPS, 1:5 mixture of AX5568 labeled LPS and unlabeled *E*. *coli* O55:B5 LPS) was added to the caspase-4 immobilized surface. The chamber was washed with buffer G, and both caspase-4 (C258A)-EGFP (or Δ80-Caspase-4 (C258A)-EGFP) and the captured LPS were analyzed. For the imaging of single-molecule co-localization, the-objective type TIRF microscope was utilized. Caspase-4 (C258A)-EGFP or (Δ80 Caspase-4 (C258A)-EGFP and AX568-LPS was excited with a 488 nm and 532 nm diode-pumped laser (Coherent, USA), respectively. The fluorescence was collected by 60X oil-immersion TIRF objective (NA 1.48), and a dichroic beamsplitter (Di01-R405/488/532/635, Semrock, USA) and notch filter (NF03-405/488/532/635, Semrock, USA) was used to remove excitation beam. Caspase-4 (C258A)-EGFP was imaged for the first 50 frames with a 0.1 s integration time, and the excitation beam was changed to image AX568-LPS for the next 50 frames. Images were recorded by an EMCCD (Andor Ixon, DU897ECS). The relative localization of EGFP and AX568 spots was manually analyzed using ImagePro software (Media Cybernetics, USA). The first 10 frames were added together to determine an appropriate threshold, and the number of fluorescent spots from the averaged images was counted. When two spots overlapped 85% at least, they were considered co-localized.

## Electronic supplementary material

Supplementary information (Supplementary Figure S1–S8)

## Data Availability

The data supporting the findings of the study are included in the paper along with its Supplementary Information, and are also available from the corresponding author upon request.

## References

[1] Raetz CR, Whitfield C (2002). Lipopolysaccharide endotoxins. Annual review of biochemistry.

[2] Tobias PS, Soldau K, Gegner JA, Mintz D, Ulevitch RJ (1995). Lipopolysaccharide binding protein-mediated complexation of lipopolysaccharide with soluble CD14. J Biol Chem.

[3] Park BS (2009). The structural basis of lipopolysaccharide recognition by the TLR4–MD-2 complex. nature.

[4] Ryu J-K (2017). Reconstruction of LPS Transfer Cascade Reveals Structural Determinants within LBP, CD14, and TLR4-MD2 for Efficient LPS Recognition and Transfer. Immunity.

[5] Poltorak A (1998). Defective LPS signaling in C3H/HeJ and C57BL/10ScCr mice: mutations in Tlr4 gene. Science.

[6] Hagar JA, Powell DA, Aachoui Y, Ernst RK, Miao EA (2013). Cytoplasmic LPS activates caspase-11: implications in TLR4-independent endotoxic shock. Science.

[7] Kayagaki N (2013). Noncanonical inflammasome activation by intracellular LPS independent of TLR4. Science.

[8] Shi J (2014). Inflammatory caspases are innate immune receptors for intracellular LPS. Nature.

[9] Kayagaki N (2015). Caspase-11 cleaves gasdermin D for non-canonical inflammasome signalling. Nature.

[10] Vanaja SK (2016). Bacterial outer membrane vesicles mediate cytosolic localization of LPS and caspase-11 activation. Cell.

[11] Pilla DM (2014). Guanylate binding proteins promote caspase-11–dependent pyroptosis in response to cytoplasmic LPS. Proceedings of the National Academy of Sciences.

[12] Meunier E (2014). Caspase-11 activation requires lysis of pathogen-containing vacuoles by IFN-induced GTPases. Nature.

[13] Wacker MA, Teghanemt A, Weiss JP, Barker JH (2017). High-affinity caspase-4 binding to LPS presented as high molecular mass aggregates or in outer membrane vesicles. Innate Immunity.

[14] Mamat, U. *et al*. Endotoxin-free protein production [mdash] ClearColi [trade] technology. *Nature Methods* **10** (2013).

[15] Dumon-Seignovert L, Cariot G, Vuillard L (2004). The toxicity of recombinant proteins in Escherichia coli: a comparison of overexpression in BL21(DE3), C41(DE3), and C43(DE3). Protein Expr Purif.

[16] The UniProt, C (2017). UniProt: the universal protein knowledgebase. Nucleic Acids Res.

[17] Sasaki H, White SH (2008). Aggregation behavior of an ultra-pure lipopolysaccharide that stimulates TLR-4 receptors. Biophysical journal.

[18] Lebrun L, Junter GA (1994). Diffusion of Dextran through Microporous Membrane Filters. J Membrane Sci.

[19] Flood B (2015). Altered expression of caspases‐4 and‐5 during inflammatory bowel disease and colorectal cancer: Diagnostic and therapeutic potential. Clinical & Experimental Immunology.

[20] Santos, J. C. *et al*. LPS targets host guanylate-binding proteins to the bacterial outer membrane for non-canonical inflammasome activation. *EMBO J*, 10.15252/embj.201798089 (2018). 10.15252/embj.201798089 PMC5852652 29459437

[21] Komatsu, T. *et al*. Disaggregation of lipopolysaccharide by albumin, hemoglobin or high-density lipoprotein, forming complexes that prime neutrophils for enhanced release of superoxide. *Pathog Dis* **74**, 10.1093/femspd/ftw003 (2016). 10.1093/femspd/ftw003 26772654

[22] Chung C, Niemela SL, Miller RH (1989). One-step preparation of competent Escherichia coli: transformation and storage of bacterial cells in the same solution. Proceedings of the National Academy of Sciences.

[23] Kim SH (2017). Reversible Regulation of Enzyme Activity by pH-Responsive Encapsulation in DNA Nanocages. ACS Nano.

