# Supplementary material for: Caspase-4 disaggregates lipopolysaccharide micelles via LPS-CARD interaction
Source: Sci Rep. 2019 Jan 29;9:826. doi: 10.1038/s41598-018-36811-4 (PMC6351570; doi:10.1038/s41598-018-36811-4)
Supplement: Supplementary file 1 — Supplementary information (Supplementary Figure S1–S8) [file 41598_2018_36811_MOESM1_ESM.pdf]

Supplementary Information

Caspase-4 disaggregates lipopolysaccharide micelles via LPS-CARD interaction

Jinsu An[1,2], Seong Ho Kim[1,2,] Dohyeon Hwang[1,2], Kyung Eun Lee[3], Min Jung Kim[4], Eun Gyeong Yang[1], So Yeon Kim[1,2], and Hak Suk Chung[1,2]*

[1]Center for Theragnosis, Biomedical Research Institute, Korea Institute of Science and Technology, Seoul 02792, Republic of Korea; [2]Division of Bio-Medical Science & Technology, KIST School, Korea University of Science and Technology, Seoul 02792, Republic of Korea; [3]Advanced Analysis Center, Korea Institute of Science and Technology, Hwarangno 14-gil 5, Seongbuk-gu, Seoul 02792, Republic of Korea; [4]Department of Biological Sciences, Sookmyung Women's University, Seoul, Korea

Running title: Caspase-4 breaks large LPS micelles to smaller complexes

*To whom correspondence should be addressed: Hak Suk Chung: Center for Theragnosis, Biomedical Research Institute, Korea Institute of Science and Technology, Seoul 02792, Republic of Korea; hschung@kist.re.kr; Tel. +82-2-958-6423; Fax. +82-2-958-5909.

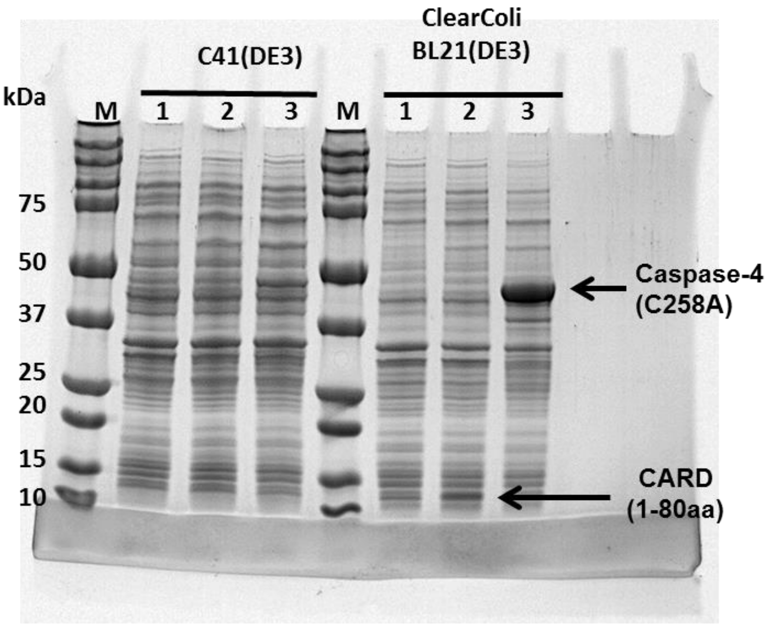

Supplementary Figure S1. Full-length SDS-PAGE gel corresponding to Fig. 2a

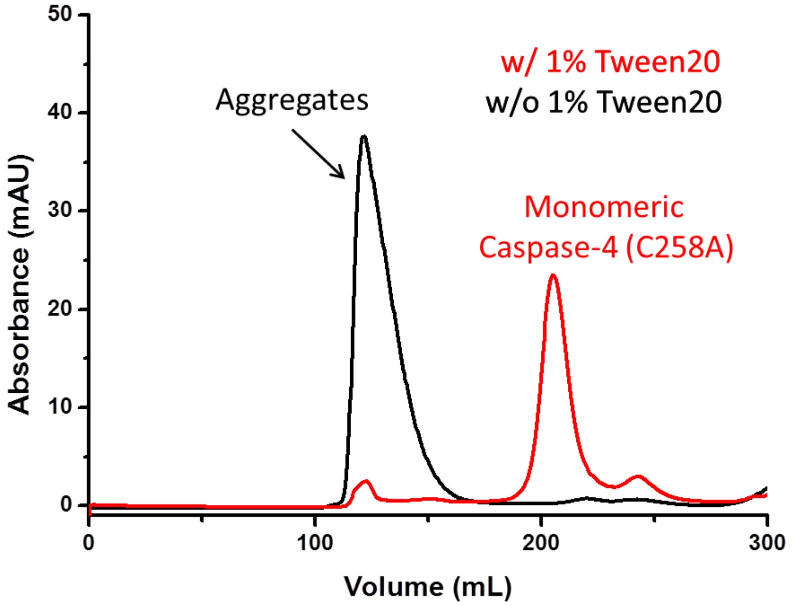

Supplementary Figure S2. SEC elution profiles of caspase-4 (C258A) purified in the presence of 1% tween 20 (Red line) or in the absence of tween 20 (Black line) in the lysis step.

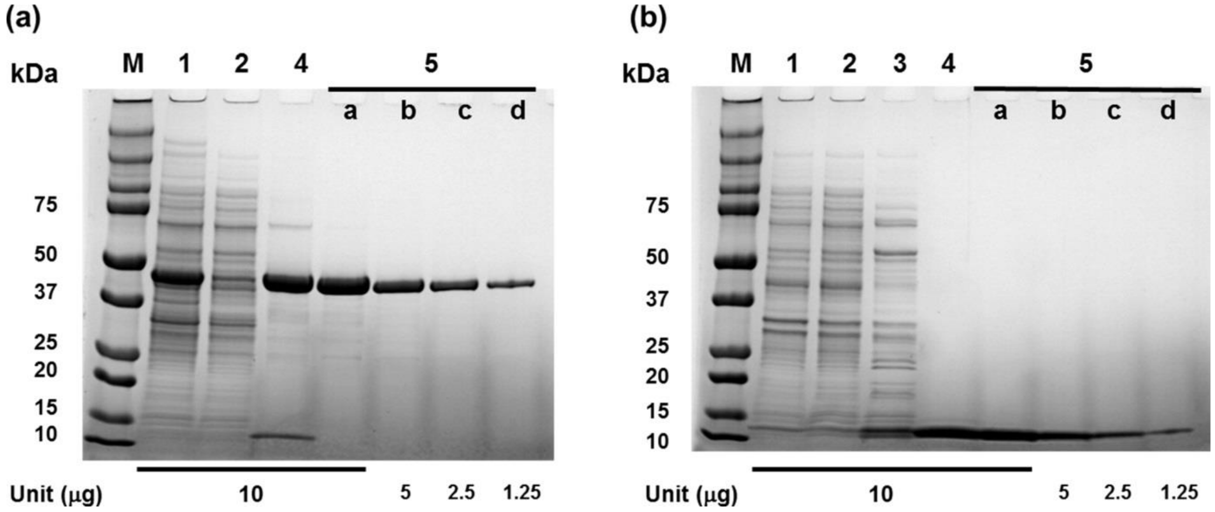

Supplementary Figure S3. Purification of caspase-4 (C258A) (a) and CARD domain (b). SDS-PAGE analysis of 10 μg of protein samples from each step of protein purification except 5b, 5c, and 5d. Protein marker (M), whole cell lysates (Lane 1), soluble fraction (Lane 2), heat treated sample (Lane 3), Ni-affinity chromatography (Lane 4), and size exclusion chromatography (Lane 5), 5 μg (Lane 5b), 2.5 μg (Lane 5c), and 1.25 μg (Lane 5d) of purified proteins.

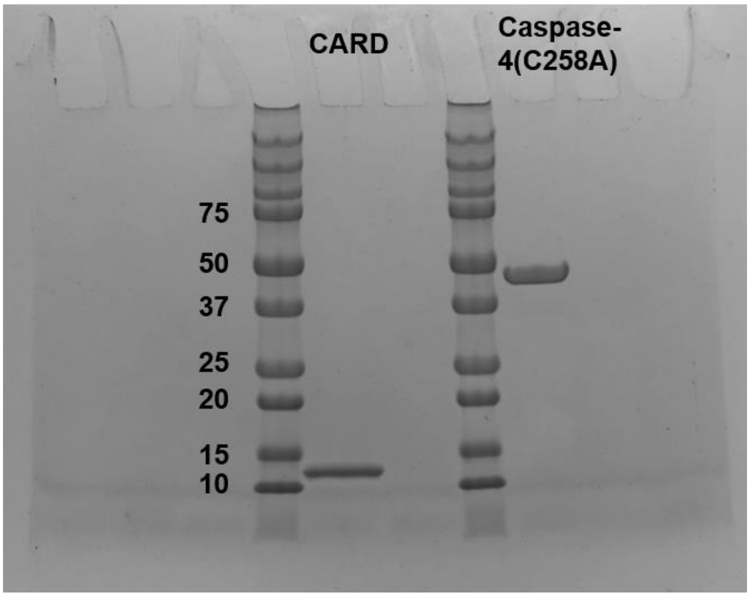

Supplementary Figure S4. Full-length SDS-PAGE gel corresponding to the insets of Fig. 2b and 2c. 5 μg of purified protein was loaded on each lane.

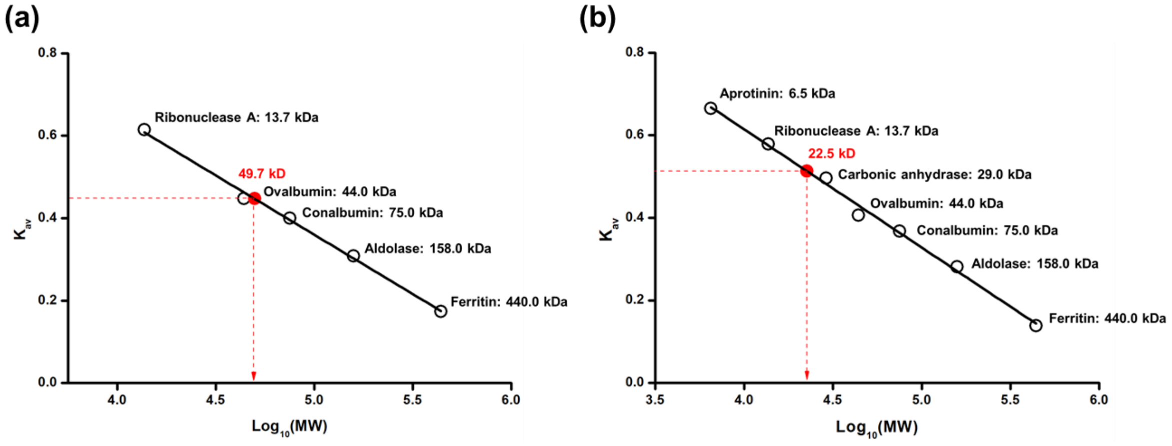

Supplementary Figure S5. Calibration curves for SEC shown in Fig. 2b (a) and 2c (b).

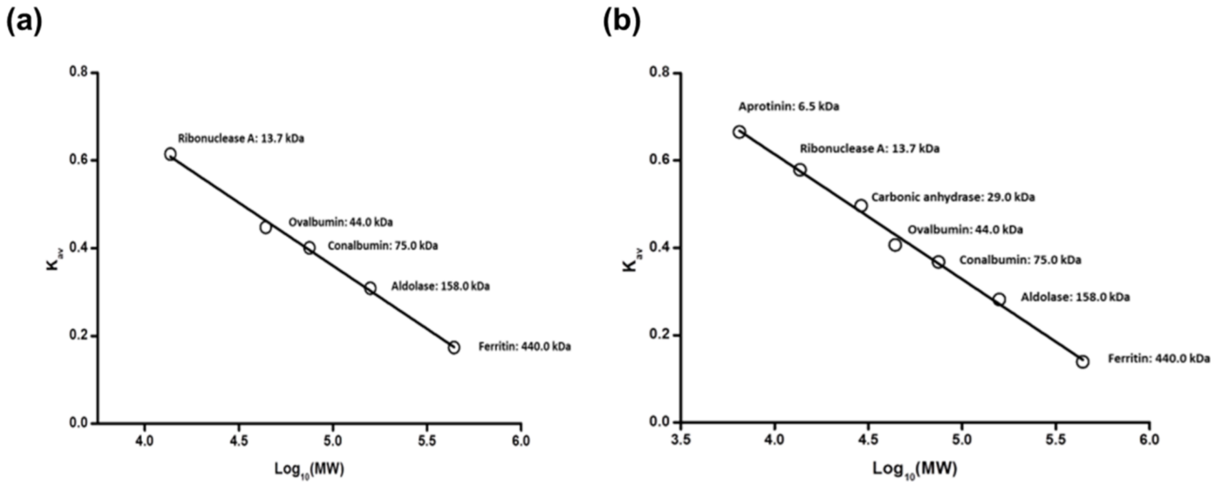

Supplementary Figure S6. Calibration curves for SEC shown in Fig. 3a and 3b (a) and 3c (b).

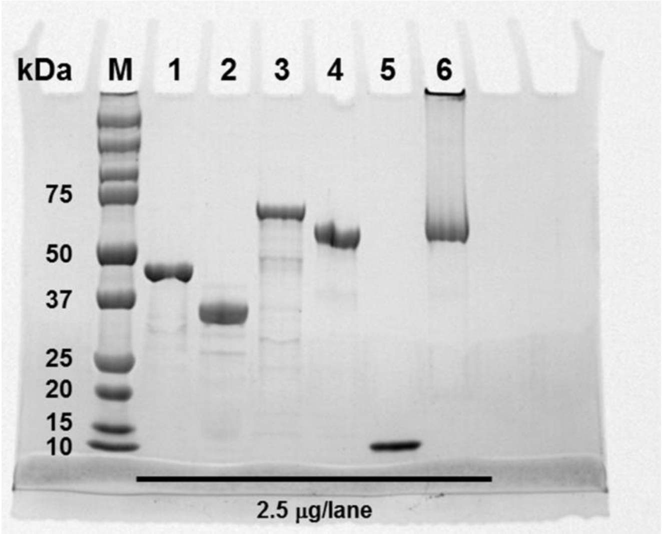

Supplementary Figure S7. A SDS-PAGE gel of the purified proteins. 2.5 μg of His-caspase-4 (C258A) (Lane 1), His-Δ80 caspase-4 (C258A) (Lane 2), His-caspase-4 (C258A)-EGFP (Lane 3), His-Δ80 caspase-4 (C258A)-EGFP (Lane 4), His-CARD (1-80aa) (Lane 5), and Human LBP-His Tag (Lane 6) were loaded on each well. Predicted molecular weights are 45.4, 36.0, 73.1, 63.8, 11.6 and 62.0 (including glycosylation) kDa, respectively.

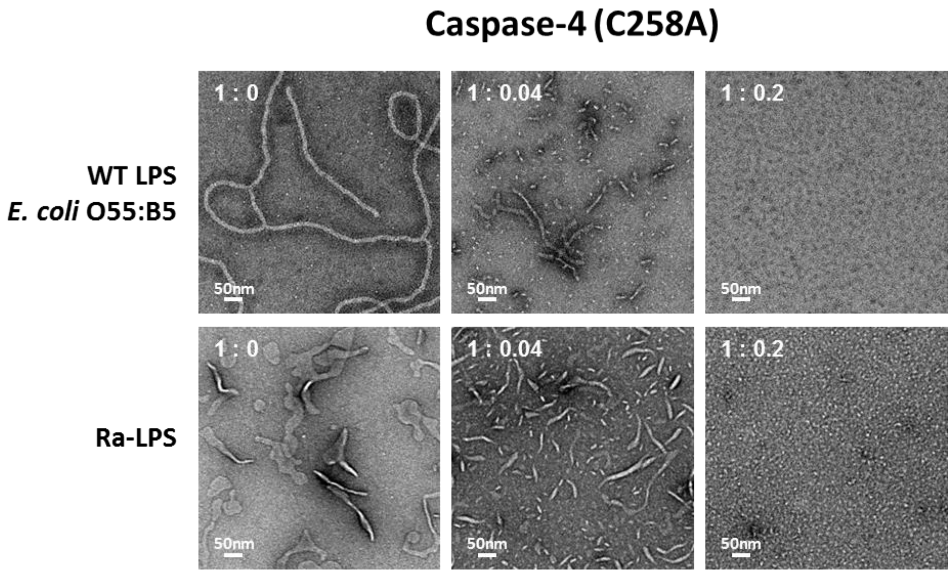

Supplementary Figure S8. Caspase-4 (C258A) disaggregates both WT-LPS and Ra-LPS micelles to smaller LPS/protein complexes. TEM images of the different molar ratios of WT-LPS/caspase-4 (C258A) (a) and Ra-LPS/caspase-4 (C258A) (b).
